# Isotretinoin Oil-Based Capsule Formulation Optimization

**DOI:** 10.1155/2013/856967

**Published:** 2013-08-26

**Authors:** Pi-Ju Tsai, Chi-Te Huang, Chen-Chou Lee, Chi-Lin Li, Yaw-Bin Huang, Yi-Hung Tsai, Pao-Chu Wu

**Affiliations:** ^1^Department of Business Administration, I-Shou University, No. 1, Section 1, Syuecheng Road, Dashu Township, Kaohsiung County 840, Taiwan; ^2^School of Pharmacy, Kaohsiung Medical University, 100 Shih-Chuan 1st Road, Kaohsiung City 80708, Taiwan; ^3^Graduate Institute of Clinical Pharmacy, Kaohsiung Medical University, 100 Shih-Chuan 1st Road, Kaohsiung City 80708, Taiwan

## Abstract

The purpose of this study was to develop and optimize an isotretinoin oil-based capsule with specific dissolution pattern. A three-factor-constrained mixture design was used to prepare the systemic model formulations. The independent factors were the components of oil-based capsule including beeswax (*X*_1_), hydrogenated coconut oil (*X*_2_), and soybean oil (*X*_3_). The drug release percentages at 10, 30, 60, and 90 min were selected as responses. The effect of formulation factors including that on responses was inspected by using response surface methodology (RSM). Multiple-response optimization was performed to search for the appropriate formulation with specific release pattern. It was found that the interaction effect of these formulation factors (*X*_1_ *X*_2_, *X*_1_ *X*_3_, and *X*_2_ *X*_3_) showed more potential influence than that of the main factors (*X*_1_, *X*_2_, and *X*_3_). An optimal predicted formulation with *Y*_10 min_, *Y*_30 min_, *Y*_60 min_, and *Y*_90 min_ release values of 12.3%, 36.7%, 73.6%, and 92.7% at *X*_1_, *X*_2_, and *X*_3_ of 5.75, 15.37, and 78.88, respectively, was developed. The new formulation was prepared and performed by the dissolution test. The similarity factor *f*_2_ was 54.8, indicating that the dissolution pattern of the new optimized formulation showed equivalence to the predicted profile.

## 1. Introduction

The development of a pharmaceutical product is a complex task because the optimal formulation involves many raw excipients and process variables, and, in the meanwhile, it must meet at least one requirement (responses) such as hardness, disintegration, and the dissolution rates at specific times. In past decades, this task has been achieved through trial and error requiring great expertise and experience, which are time-consuming and expensive. Furthermore, it is difficult to determine when and whether the optimal process or composition of excipients has been actually obtained. Recent studies [1–8] demonstrated that the response surface methodology (RSM) including statistical experimental design such as Box-Behnken design, central composite design, factorial design, and mixture design, along with multiple linear regression analysis is a useful and reliable method to overcome the shortcomings of traditional methods. The procedure of optimization method involves (1) systemic/statistical formulation design to minimize the number of experiments and (2) an RSM to analyze the relationships between response variables and causal variables via a set of constrained equations and to obtain an optimal formulation [9].

3,7-Dimethyl-9-(2,6,6-trimethyl-1-cyclohexen-1-yl)2-cis-4-trans-6-trans-8-trans-nonatetraenoic acid (13-cis-retinoic acid, isotretinoin) has been introduced to treat severe recalcitrant acne by oral administration since 1982, and, until now, it was the only compound capable of curing the disease [10–12]. The full mechanism of action of isotretinoin on treatment of acne is unknown. Recent studies demonstrated that isotretinoin induces apoptosis in human sebaceous glands and in sebocytes both *in vitro* and *in vivo* by enhancing the expression of neutrophil gelatinase-associated lipocalin and then results in the reduction of the size and secretion of the human sebaceous gland [13, 14]. 

In this present study, the isotretinoin was used as a model drug to develop an oil-based capsule. The RSM with mixture design has been proposed to evaluate the influence of individual factors and their interactive effects on the dissolution pattern and to obtain an optimal formulation with desirable dissolution rate.

## 2. Materials and Methods

### 2.1. Materials

Isotretinoin and retinoic acid were purchased from Sigma-Aldrich (St Louis, MO, USA). Hydrogenated coconut oil and soybean oil were acquired from Showa Corporation (Saitama, Japan). Yellow beeswax was purchased from Tokyo Chemical Industry (Tokyo, Japan). Roaccutane capsule containing isotretinoin 10 mg was purchased from Roche (Eberbach, Germany) in the Taiwan Market. All other chemicals and solvents were of analytical reagent grade.

### 2.2. Preparation of Isotretinoin Capsules

A computer-generated experimental design with constrained mixture design [9] was built to evaluate the effects of the formulation factors on the dissolution rate of drug from oil-based capsules. The concentration of yellow wax (*X*_1_), hydrogenated coconut oil (*X*_2_), and soybean oil (*X*_3_) was included as independent variables. The variables and their variation levels are listed in Table 1. The range of the variation levels was set according to our preliminary data. The total amount of isotretinoin oil-based formulation was 160 mg composed of drug 10 mg, vitamin E 2 mg, and different amounts of yellow wax, hydrogenated coconut oil, and soybean oil. The detailed composition of these model formulations is presented in Table 2.

The yellow wax, hydrogenated coconut oil, soybean oil, and drug were placed in a beaker and heated to 70°C in a dark room for 15 min. The mixture of 160 mg was deposited into a capsule by a dropper when it was well mixed. The capsules were stored in light resistant container.

### 2.3. Determination of the Dissolution Rate of Isotretinoin from Oil-Based Capsule

The basket method of United States Pharmacopoeia (USP 34) [15] was used for the dissolution studies. Nine hundred milliliters of 0.05 M phosphate buffer, pH 7.8, containing 0.5% w/v N-N-dimethyldodecylamine N-oxide of 900 mL, was used as a dissolution medium [15] and was maintained at 37°C. The rate of stirring was 100 rpm. At specific intervals, five milliliters of samples was withdrawn. The samples were analyzed by HPLC. Six capsules of each formulation were determined. 

### 2.4. HPLC Analysis

HPLC analysis was carried out with a Hitachi L-7100 series HPLC system. A LiChrospher RP-18 column (250 × 4.0 mm I.D., particle size 3 *μ*m) was used. The mobile phase consisted of methanol and 2.5 mM monobasic potassium phosphate (adjusted to pH 2.1 by phosphoric acid) at the ratio of 81 : 19, at the flow rate of 1.5 mL/min. The detection wave was at 358 nm. Tretinoin of 20 *μ*g/mL was used as internal standard. The concentration range of the isotretinoin was found to have linearity from 0.1 to 10 *μ*g/mL (*r* ^2^ = 0.9995). The low of quantitation was 0.08 *μ*g/mL.

### 2.5. Data Analysis

The drug release percentages at 10, 30, 60, and 90 min (responses) and the code values of independent variables of all systemic capsules were treated with Design-Expert software. The suitable polynomial equations for mixture design consisting of three variables included linear, quadratic, and special cubic models. The terms were significant when the *P* value was less than 0.05. The best fitting mathematical equation was chosen based on the comparisons of some statistical parameters including the *P* value of the model, the multiple correlation coefficient (*R* ^2^), adjusted multiple correlation coefficient (adjusted *R* ^2^), and the coefficient of variation (C.V.) validated by Design-Expert software [16, 17].

The Korsmeyer-Peppas' equation was used to broach a possible release mechanism of isotretinoin from oil-based capsule [18, 19]:

(1) $$\begin{array}{c} \frac{M_{t}}{M_{\infty }}=kt^{n}, \end{array}$$

where *M*_*t*_ is the drug release percentage at time *t*; *M*_*∞*_ is the release percentage after infinite time, usually taken as 100; *M*_*t*_/*M*_*∞*_ is the fraction release percentage at time *t*; *k* is a release constant associated to the properties of the oil-based system. The value of *n* is the release exponent, indicative of the drug release mechanism. A value of *n* = 0.45 demonstrates Fickian diffusion, 0.45 < *n* < 0.89 reveals anomalous transport, and *n* > 1.0 demonstrates super case-II transport.

The similarity factor *f*_2_ was used to compare the difference between the experimental formulation and the control group [20, 21]:

(2) $$\begin{array}{c} f_{2}=50\times \log\left\{{\left[1+\left(\frac{1}{n}\right)\sum_{t=1}^{n}{\left(Rt-Tt\right)}^{2}\right]}^{-0.5}\times 100\right\}, \end{array}$$

where *Rt* and *Tt* are the percentages of isotretinoin dissolved at each time point, *t*, for the test and reference dissolution profiles, respectively. The *n* is the number of samples. The value of *f*_2_ greater than 50 (50–100) indicates equivalence or sameness of the two dissolution curves. 

## 3. Results and Discussion

**Figure 1:**
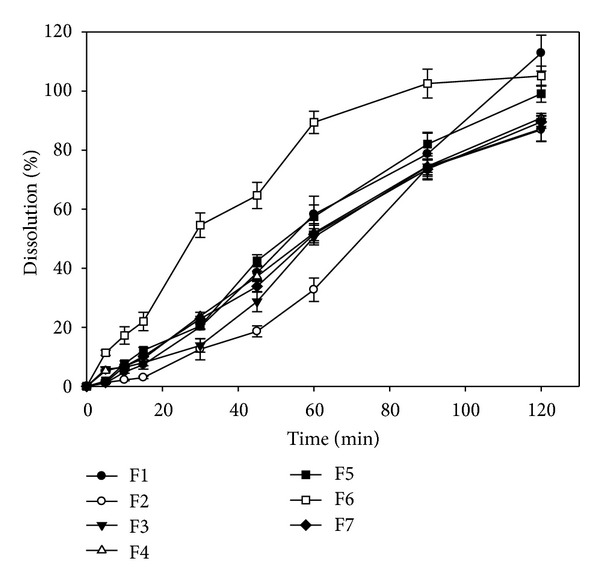
Dissolution profiles of isotretinoin oil-based capsules.

The dissolution patterns of all isotretinoin capsules are shown in Figure 1. It can be seen that the seven model formulations with different proportions of yellow wax/hydrogenated coconut oil/soybean oil showed different dissolution patterns. The Korsmeyer-Peppas' equation [18, 19] was used to explore the release mechanism. As shown in Table 2, all patterns of formulations can be described by the Korsmeyer-Peppas' equation (*r* > 0.9336). The diffusional exponent of all formulations ranged from 0.83 to 1.47, indicating that the release mechanism of drug from oil-based matrix belonged to case-II transport [18, 19]. The release rate ranged from 0.09 to 2.78, demonstrating that the release rates were significantly influenced by the formulation factor combinations. 

According to the U.S.P. monographs [15] for the isotretinoin capsule, the drug release percentage in 90 min must be greater than 80%. In this study, only formulations 5 and 6 met the requirement. In comparison to the dissolution rate between formulation 5 and formulation 6, formulation 6 with higher amount of wax and hydrogenated coconut oil showed higher release rate at each time point than that of formulation 5. The melting points of beeswax, hydrogenated coconut oil, and soybean oil were 62~64°C and 36~40°C, respectively, and higher than room temperature [22]. The result indicated that the melting point of ingredients was not the major determinant for drug release from the oil-based matrix. The affinity of drug and excipients might dominate the release rate. 

**Figure 2:**
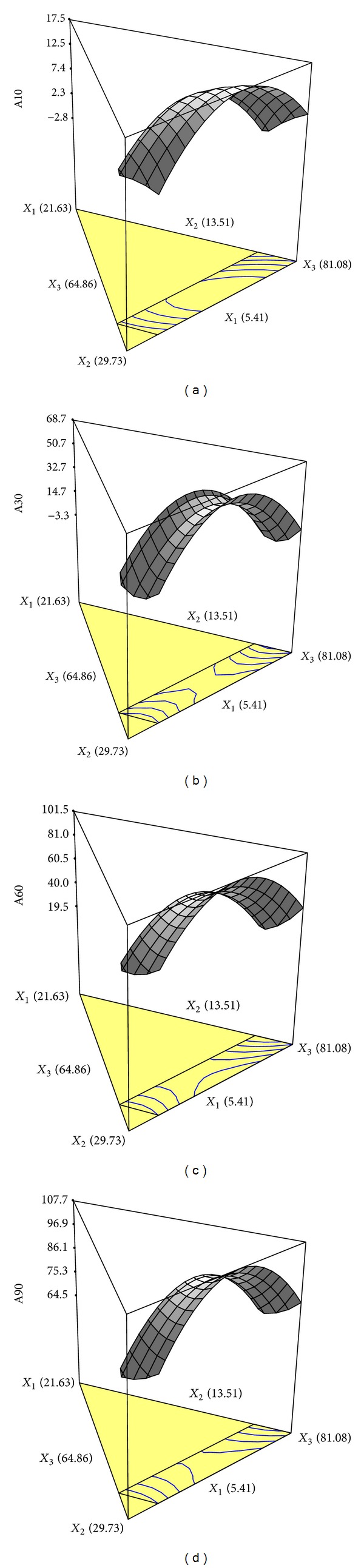
The three-dimensional contour diagrams illustrating the effect of beeswax (*X*_1_), hydrogenated coconut oil (*X*_2_), and soybean oil (*X*_3_) on the isotretinoin release percentage at 10 min (a), 30 min (b), 60 min (c), and 90 min (d).

In order to realize the effect of independent formulation factors on the drug release profiles, the RSM [5–7] was used. The drug release percentages at 10, 30, 60, and 90 min were selected as responses (*Y*_10 min⁡_, *Y*_30 min⁡_, *Y*_60 min⁡_, and *Y*_90 min⁡_), to recognize the burst effect in the earlier stage and assure that most of the isotretinoin was released over time. After polynomial linear regression, the result of statistical parameters is summarized in Table 3. The obtained coefficient of determination demonstrated that more than 90% of the dependent variations (responses) of release percentages at 10, 30, 60, and 90 min could be described by mixture quadratic equations as the function of the formulation factors. A large *F*-value and a small *P* value (*P* < 0.05) indicate a significant effect on the respective response variables. In the regression equation, a positive value of coefficient represents an effect that favors the optimization due to synergistic effect, when a negative value demonstrates an antagonistic effect or inverse relationship between the independent factor and the response [5–7]. In terms of the main factors, the beeswax (*X*_1_) showed the most effect. The result might be due to the beeswax with highest melting point from 62 to 64°C, resulting in the solidification of the oil-based mixture which may be one of the retardants for the oil-based capsule. The effect of hydrogenated soybean oil (*X*_2_) and soybean oil (*X*_3_) was similar. In terms of the interaction factors, it was found that the interaction effect of these formulation factors (*X*_1_ *X*_2_, *X*_1_ *X*_3_, and *X*_2_ *X*_3_) showed more potential influence than that of the individual main factors (*X*_1_, *X*_2_, and *X*_3_). In the early stage (*Y*_10 min⁡_), the interaction effect of *X*_1_ *X*_2_ showed the most effect followed by *X*_1_ *X*_3_ and *X*_2_ *X*_3_, indicating the melting point of excipients was the important influencing factor at this stage. In the later stage, the interaction effect of *X*_1_ *X*_3_ showed the most effect. The three dimensional response surface plots of *Y*_10 min⁡_, *Y*_30 min⁡_, *Y*_60 min⁡_, and *Y*_90 min⁡_ (Figure 2) also showed that the effect of formulation factors in the early stage was different to the later stages. In the later stage, formulations with low level of *X*_1_ and medium level of *X*_2_ and *X*_3_ showed higher release percentages.

**Figure 3:**
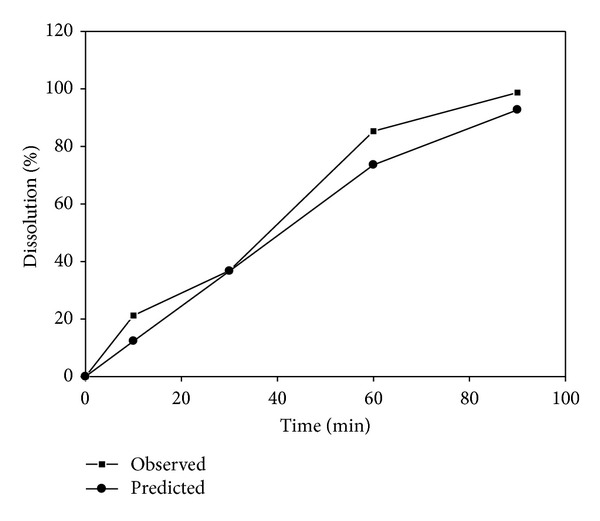
Dissolution profiles of predicted and observed isotretinoin capsules.

In order to obtain an optimal formulation with an adequate release percent at different time points, the commercial product was used as control. According to the release pattern of the commercial product, the range of responses were restricted to 0% < *Y*_10 min⁡_ < 15%; 15% < *Y*_30 min⁡_ < 40%; 70% < *Y*_60 min⁡_ < 85%; and 85% < *Y*_90 min⁡_ < 100% (Table 1). Under these conditions, the model predicted a new formulation with *Y*_10 min⁡_, *Y*_30 min⁡_, *Y*_60 min⁡_, and *Y*_90 min⁡_ values of 12.3%, 36.7%, 73.6%, and 92.7% at *X*_1_, *X*_2_, and *X*_3_ values of 5.75, 15.37, and 78.88, respectively. To verify these values, the new formulation was prepared and subjected to the dissolution test. The predicted profile and the dissolution profiles of the new formulation are shown in Figure 3. Both dissolution curves were compared by using the similarity factor (*f*_2_) [20, 21]. The *f*_2_ value was 54.8, which is greater than the criteria value of 50, showing an equivalence to the predicted profile and the release profile of the optimal formulation. Furthermore, the *f*_2_ value of comparison of the commercial product and the experimental formulation was 56.7, indicating that the release profile of the experimental formulation was similar to the commercial product.

## 4. Conclusion 

It was concluded that RSM and multiple-response optimizations utilizing a polynomial equation can be successfully used to design an isotretinoin oil-based formulation for a predetermined release profile.

## Figures and Tables

**Table 1:** Summary of variables for the mixture design.

Causal factors variables	Code (amount)	
	Low	High
X_1_: beeswax	0 (8)	0.166 (12)
X_2_: hydrogenated coconut oil	0 (20)	0.834 (40)
*X*_3_: soybean oil	0 (96)	1.000 (120)

Response variables	Constraints	

*Y*_10 min⁡⁡_: percent dissolved in 10 min	0% ≤ *Y*_1_ ≤ 15%	
*Y*_30 min⁡⁡_: percent dissolved in 4 h	15% ≤ *Y*_2_ ≤ 40%	
*Y*_60 min⁡⁡_: percent dissolved in 8 h	70% ≤ *Y*_3_ ≤ 85%	
*Y*_90 min⁡⁡_: percent dissolved in 14 h	85% ≤ *Y*_4_ ≤ 100%	

(1) The total amount of isotretinoin oil-based capsule was 160 mg composed of isotretinoin 10 mg, vitamin E 2 mg, and other ingredients 148 mg including beeswax (*X*_1_), hydrogenated coconut oil (*X*_2_), and soybean oil (*X*_3_).

(2) *X*_1_ + *X*_2_ + *X*_3_ = 148 mg  =  100%.

**Table 2:** The composition and dissolution parameters of isotretinoin oil-based capsules.

	*X*_1_		*X*_2_		*X*_3_		*K*	*n*	*R*	*Y*_10 min⁡_	*Y*_30 min⁡_	*Y*_60 min⁡_	*Y*_90 min⁡_
	Code	mg	Code	mg	Code	mg				%	%	%	%
F1	0.000	8	0.834	40	0.166	100	0.13	1.47	0.9920	4.9	20.1	58.2	78.7
F2	0.166	12	0.000	20	0.834	116	0.09	1.44	0.9781	2.2	12.6	32.7	74.1
F3	0.083	10	0.834	40	0.083	98	0.83	0.95	0.9336	6.4	13.8	50.6	74.4
F4	0.166	12	0.834	40	0.000	96	0.81	0.99	0.9831	6.8	23.8	51.9	74.5
F5	0.000	8	0.000	20	1.000	120	0.33	1.26	0.9789	7.7	20.4	57.4	82.0
F6	0.083	10	0.417	30	0.500	108	2.78	0.83	0.9954	17.2	54.6	89.4	102.5
F7	0.166	12	0.834	40	0.000	96	0.23	1.32	0.9823	6.7	22.8	51.5	73.5

(1) The total amount of isotretinoin oil-based capsule was 160 mg composed of drug 10 mg, vitamin E 2 mg, and other ingredients including beeswax (*X*_1_), hydrogenated coconut oil (*X*_2_), and soybean oil (*X*_3_) 148 mg.

(2) Release mechanism fitted by Korsmeyer-Peppas' equation: *M*_*t*_/*M*_*∞*_ = *kt* ^*n*^, *k*, *n*, and *r* are release rate, diffusion exponent, and correlation coefficient.

(3) Y_*i*_: responses, the drug release percentage at 10 min (*Y*_10 min⁡_), 30 min (*Y*_30 min⁡_), 60 min (*Y*_60 min⁡_), and 90 min (*Y*_90 min⁡_).

(4) The composition of each run was random and arranged according to the D-optimal model proved Design-Expert software.

**Table 3:** Optimal regression equation for each response variable.

	*Y*_10 min⁡_	*Y*_30 min⁡_	*Y*_60 min⁡_	*Y*_90 min⁡_
*b*_1_(*X*_1_)	−100.04	922.67	436.82	272.32
*b*_2_(*X*_2_)	−6.45	−21.46	16.45	51.87
*b*_3_(*X*_3_)	7.67	20.39	57.42	82.03
*b*_12_(*X*_1_ *X*_2_)	207.53	−809.95	−250.14	−104.70
*b*_13_(*X*_1_ *X*_3_)	89.44	−1138.72	−633.23	−285.57
*b*_23_(*X*_2_ *X*_3_)	64.65	249.62	251.99	157.24

*F*	17909.89	499.69	4092.21	264.78
*P*	0.0057	0.0340	0.0119	0.0466
C.V.	0.52	2.88	0.52	0.88
*R* ^2^	1.0000	0.9996	1.0000	0.9992
Adjusted *R* ^2^	0.9999	0.9976	0.9997	0.9955
